# Association between cardiovascular health and epigenetic aging: a twin study

**DOI:** 10.1186/s13148-025-02034-4

**Published:** 2026-01-05

**Authors:** Hui Cao, Ziyun Jiang, Weihua Cao, Jun Lv, Canqing Yu, Tao Huang, Dianjianyi Sun, Chunxiao Liao, Yuanjie Pang, Runhua Hu, Ruqin Gao, Min Yu, Jinyi Zhou, Xianping Wu, Yu Liu, Wenjing Gao, Liming Li

**Affiliations:** 1 Department of Epidemiology and Biostatistics, School of Public Health, Peking University, Beijing, China; 2 Key Laboratory of Epidemiology of Major Diseases (Peking University), Ministry of Education, Beijing, China; 3 Qingdao Center for Disease Control and Prevention, Qingdao, China; 4 Zhejiang Center for Disease Control and Prevention, Hangzhou, China; 5 Jiangsu Center for Disease Control and Prevention, Nanjing, China; 6 Sichuan Center for Disease Control and Prevention, Chengdu, China; 7 Heilongjiang Center for Disease Control and Prevention, Harbin, China

**Keywords:** Life’s essential 8, Epigenetic clock, Cardiovascular health, Twin study

## Abstract

**Objective:**

To explore the association between life’s essential 8 and epigenetic age based on twins population.

**Methods:**

This study included 1030 twins (515 pairs) for cross-sectional analysis and conducted cross-lagged analysis among 294 twins (147 pairs) who participated in both the baseline and follow-up surveys from the Chinese National Twin Registry. LE8 scores were obtained from measurements based on American Heart Association definitions. DNA methylation data were used to calculate epigenetic age metrics, including GrimAA, DamAA and DunedinPACE. Linear mixed-effect models were applied for cross-twin analyses and within-monozygotic-pair analyses.

**Results:**

In the cross-sectional analysis, higher LE8 score was associated with slower epigenetic aging (DunedinPACE and DamAA) in both across-twin analyses and within-monozygotic-pair analyses. In stratified analyses, the association between LE8 score and epigenetic age appeared more significant in males and in individuals aged 50 years older. The cross-lagged analysis further revealed significant temporal associations between LE8, health factor, and DunedinPACE.

**Conclusion:**

Higher LE8 scores were associated with a deceleration in biological aging.

**Supplementary Information:**

The online version contains supplementary material available at 10.1186/s13148-025-02034-4.

## Background

The American Heart Association introduced Life’s Essential 8 (LE8) [1], a comprehensive framework encompassing four health behaviors (diet, physical activity, nicotine exposure, and sleep) and four health factors (body mass index (BMI), blood lipids, blood glucose, and blood pressure), aiming to help individuals maintain cardiovascular health (CVH) and live longer, healthier lives [2].

Aging refers to a series of functional declines that occur with advancing age [3]. Aging imposes an unbearable burden of chronic disease [4], resulting in substantial societal and economic burdens. Slowing the process of biological aging can help to delay aging-related diseases, and reduce mortality risk, thereby extending both life expectancy and health span. DNA methylation [3, 5] serves as a dynamic indicator of human aging-related physiological changes, thus finding application in the estimation of biological age called epigenetic clock [6]. Current epigenetic clocks can be categorized into four generations [7]. First generation clocks are exclusively trained to predict chronological age, while next-generation clocks are designed to estimate age in ways that significantly associate with health, lifestyle, and/or aging-related outcomes [7]. The GrimAge [8], a second-generation clock, includes 1030 cytosine-phosphate-guanine (CpG) sites and was developed using seven plasma proteins, smoking pack-years, and chronological age as training phenotypes. DunedinPACE [9] is a third-generation clock, consisting of 173 CpGs associated with the rate of longitudinal change in 19 biological markers over a 20-year period. The first- to third-generation epigenetic clocks are all based on correlations between CpG sites and aging-related phenotypes, and it remains unclear whether the DNA methylation differences used for age prediction are causal factors of aging-related phenotypes or are secondary consequences of the aging process. DamAge [10], a fourth-generation clock, was developed using epigenome-wide Mendelian Randomization (EWMR), constructing a causality-enriched clock. The differences between chronological age and epigenetic age (EA) are called epigenetic age acceleration (EAA), including measures such as GrimAge acceleration (GrimAA) and DunedinAge acceleration (DamAA). GrimAA and DamAA reflect the cumulative aging burden, whereas DunedinPACE estimates the current pace of aging.

The association between LE8 and epigenetic clocks has been explored in previous studies [11–14]; however, no study has yet examined the relationship between LE8 and DamAA. Compared to earlier-generation clocks, the study showed that DamAA [10] more robustly reflects the effects of age-related conditions and can also capture aging-related changes induced by short-term interventions. Investigating the association between LE8 and DamAA can validate the impact of LE8 on biological pathways of aging through a causal epigenetic clock, providing causal evidence for anti-aging interventions. Both LE8 [1] and epigenetic clocks [15] are influenced by genetic factors, which may introduce the possibility of genetic confounding when assessing associations between epigenetic age metrics and LE8. A co-twin control study can exclude genetic confounding. Based on data from the Chinese National Twin Registry (CNTR), this study aims to assess the associations of three epigenetic age metrics (GrimAA, DunedinPACE, and DamAA) with LE8 score.

## Method

### Study population and zygosity assessment

The Chinese National Twin Registry (CNTR), established in 2001, is currently the largest twin cohort in Asia. To date, CNTR has recruited 61,566 twin pairs (including multiples) from 11 provinces or cities across China [16]. In addition, two specialized investigations were carried out in 2013 and during 2017–2018, which included more detailed questionnaires, physical examinations, and fasting blood sample collection. Detailed information on CNTR is available in previous studies. The study was approved by the Biomedical Ethics Committee at Peking University (IRB00001052-13022 and IRB00001052-14021) and conducted in accordance with the Declaration of Helsinki. Written informed consent was obtained from all participants.

Participants included in this study were from the two surveys of CNTR. The detailed inclusion criteria are as follows: (1) age 18 years or older; (2) complete questionnaire survey and physical examination; (3) available blood samples. Pregnant participants were excluded. We further removed incomplete co-twin pairs if either twin lacked DNAm or phenotypic data. Finally, 1030 participants (515 twin pairs, including 367 monozygotic (MZ) and 148 dizygotic (DZ) twin pairs) were included in this study. Among these, 294 participants (176 twin pairs including 118 MZ twin pairs) had baseline and follow-up data.

Zygosity was determined through a panel comprising 59 single-nucleotide polymorphisms (SNPs) on the Illumina Infinium Methylation Chips. Twins with concordance greater than 90% in these SNPs were classified as monozygosity twins [17]. Otherwise, they were classified as dizygotic (DZ) twins.

### Measurement of LE8

The LE8 score includes four health behaviors (diet, physical activity, nicotine exposure and sleep duration) and four health factors (BMI, non-high-density (non-HDL) lipoprotein cholesterol, plasma glucose, and blood pressure) [1]. Diet, physical activity, nicotine exposure and sleep duration were assessed through self-reported questionnaires. Height, weight, and blood pressure were measured by physical examination. Non-HDL cholesterol, plasma glucose, and HbA1c were measured from fasting blood samples.

The LE8 scoring followed the American Heart Association (AHA) standards [1], wherein the diet component was quantified according to Dietary Approaches to Stop Hypertension (DASH) metrics [18], based on the frequency of intake across eight dietary categories: sodium-rich foods, fruits/vegetables, legumes, whole grains, dairy products, red/processed meats, and sugar-sweetened beverages; meanwhile, the sleep duration component assigned scores using clinically defined thresholds: 0 points for ≤ 4 h, 40 points for >4 to ≤ 6 h, 80 points for >6 to ≤ 8 h, and 100 points for >8 h.

### DNA methylation data collection and processing

Illumina Human Methylation 450 K BeadChip arrays or Illumina Infinium Methylation EPIC (850 K) BeadChip arrays were used to collect DNA methylation data from all peripheral blood samples. Only CpG sites common to both platforms were considered in the subsequent procedure, which were obtained using the “combineArrays” function of the R package *minfi* (1.40.0).

Several quality control procedures were applied to exclude probes and samples in our analyses. Probes were removed if they met one or more of the following conditions: (1) detection *P*-value > 0.05 in more than 1% of samples; (2) multihit probes; (3) annotated single nucleotide polymorphisms (SNPs) on the microarray; and (4) located on the sex chromosomes. As for sample quality control, we excluded samples that (1) detection *P*-value > 0.01; and (2) exhibited sex mismatch. The DNA methylation level of each CpG site was quantified using β-value (number of methylated sites/[number of methylated and unmethylated sites]). The quantile normalization was conducted using the “preprocessQuantile” function of the R package *minfi* (1.40.0).

### Estimation of epigenetic clock

We selected one clock from each of the second to fourth generations for analysis, specifically GrimAA, DunedinPACE, and DamAA, respectively. The GrimAge clocks were calculated using the online tool provided by UCLA; the DunedinaPACE clocks were calculated using the R package *DunedinPoAm38*; and the DamAges were calculated using the *biolearn* package in Python3.12.

Epigenetic age acceleration (EAA) values for GrimAge, DamAge (denoted as GrimAA and DamAA) were defined as the residual from linear regression models regressing epigenetic age on chronological age. The values of GrimAA and DamAA represented the rate of GrimAge, and DamAge change per calendar year. If the value of GrimAA and DamAA are > 0, it indicates an accelerated rate of epigenetic age change. The same, if the value of DunedinPACE clock > 1, it indicates a faster rate of aging. DunedinPACE was calculated using the R package *DunedinPACE*.

### Covariate assessment

We selected covariates that were previously displayed or assumed to be associated with LE8 or epigenetic ageing, including age, sex (female or male), geographic region (northern or southern), educational level (< middle school, middle school, >middle school), alcohol use (yes or no), history of chronic diseases including cardiovascular disease and stroke. All covariates were collected through self-reported questionnaires.

### Statistical analysis

#### Cross-sectional analysis

To minimize genetic confounding when assessing the associations between LE8 and epigenetic age metrics, we employed a stepwise approach consisting of two analytical strategies: (1) cross-twin analyses and (2) within-monozygotic (MZ)-pair analyses.

Linear mixed-effect models were applied for both strategies. In the cross-twin analyses, LE8 was treated as the independent variable and each epigenetic clock as the dependent variable, with twin pair ID included as random intercepts to account for within-pair correlation. Model 1 adjusted for sex, age and geographic region as fixed effects, with twin pair ID as random effects. Model 2 added educational level to Model 1, and Model 3 further included history of chronic diseases and alcohol use as fixed effects.

For the within-MZ-pair analyses, we conducted the following formula:

$$\:\mathrm{E}\left({\mathrm{Y}}_{\mathrm{i}\mathrm{j}}\right)={{\upbeta\:}}_{0}+{{\upbeta\:}}_{\mathrm{w}}({\mathrm{X}}_{\mathrm{i}\mathrm{j}}-{\stackrel{-}{\mathrm{X}}}_{\mathrm{i}})+{{\upbeta\:}}_{\mathrm{b}}{\stackrel{-}{\mathrm{X}}}_{\mathrm{i}}.$$

The $\:{\mathrm{Y}}_{\mathrm{i}\mathrm{j}}$ is the epigenetic age metric for twin j within the twin pair i, $\:{\mathrm{X}}_{\mathrm{i}\mathrm{j}}$ is the LE8 score for twin j within the twin pair i, and $\:{\stackrel{-}{\mathrm{X}}}_{\mathrm{i}}$ is the mean LE8 score for the twin pair i. $\:{{\upbeta\:}}_{\mathrm{w}}$ is the within-twinpair regression coefficient, which represents the association between epigenetic age metrics and LE8 score after controlling for genetic confounding. We further explore the association between health behavior, health factor scores with epigenetic age metrics using the same method. For sensitivity analyses, we excluded the individuals with comorbidities (including cancer and cardiovascular disease) to assess the robustness of our findings. Since MZ twins are matched for sex and age, the model did not adjust for these variables. Adjustment for other covariates was consistent with across-twins analyses.

Moreover, to explore the influence of sex and age on the association of LE8 score with epigenetic age, we conducted stratified analyses by sex (male, female), age group (50years old and ≥ 50years old) across twins. In addition, formal interaction terms (sex × LE8 and age × LE8) were included in the cross-twin models to statistically test whether the associations differed by sex or age. Due to the limitation of the sample size, stratified and interaction analysis was not repeated in the MZ twins.

#### Cross-lagged analysis

**Fig. 1:**
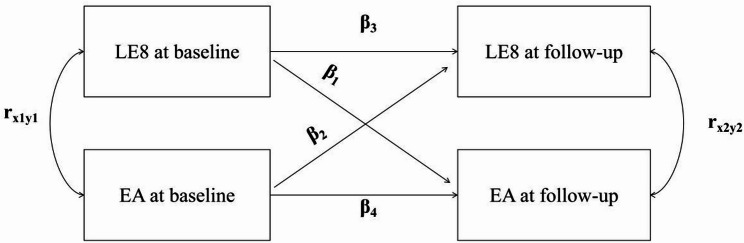
The cross-lagged panel model (CLPM). EA: epigenetic age metrics (GrimAA, DamAA, DunedinPACE); LE8: life’s essential 8. r_x1y1_, r_x2y2_: contemporaneous correlations between LE8 and EA at baseline and follow-up, respectively; β_1_, β_2_: cross-lagged effects of baseline LE8 on follow-up EA, and baseline EA on follow-up LE8, respectively; β_3_, β_4_: autoregressive effects, reflecting the stability of LE8 or EA over time.

Using longitudinal data from 294 twins, we conducted cross-lagged panel analyses via structural equation modeling to examine temporal relationships between LE8, its subscales and epigenetic age metrics. The cross-lagged panel model (CLPM) is a longitudinal analytical approach used to examine bidirectional relationships and temporal associations between two variables. As shown in Fig. 1, the CLPM decomposes the relationships between LE8 and epigenetic age metrics measured at two time points into contemporaneous covariances (r_x1y1_ and r_x2y2_, reflecting correlations between variables measured at the same time point), autoregressive effects (β_3_ and β_4_, reflecting the stability of each variable over time), and cross-lagged effects (β_1_ and β_2_, representing the predictive influence of one variable on future changes in the other). By estimating these paths simultaneously, the CLPM allows investigation of the temporal ordering and directional associations between variables while accounting for baseline correlations and stability over time.

Analyses were performed using the R package lavaan, adjusting for baseline age and sex. Twin-pair dependency was accounted for using cluster-robust standard errors with twin ID as the clustering variable. Statistical analyses for this study were carried out using R version 4.3.3 and statistical significance was ascertained by a two-sided P value of < 0.05.

## Results

### Baseline characteristics

The characteristics of the study participants are presented in Table 1. Among the 1030 participants (515 twin pairs) included in the cross-sectional analysis, mean age was 49.7 years (range: 20–82 years); 71.3%were monozygotic, and 67.2% were male. The mean LE8 score of all individuals was 68.7 (range: 35.16–96.09 points). Among 147 twin pairs with both baseline and follow-up data, 59.9% were MZ, and 60.2% were male; the mean age of the baseline and follow-up were 50.31 and 54.95 years, respectively.

Among the epigenetic age measures, both GrimAge and DamAge were highly correlated with chronological age (correlation coefficient > 0.90), whereas DunedinPACE showed a moderate correlation (correlation coefficient = 0.38). Regarding the age acceleration measures, the correlations among the age acceleration measures (GrimAA, DamAA, and DunedinPACE) were low to moderate (correlation coefficient = 0.13 to 0.36), with DamAA demonstrating the lowest correlations with the other measures (Figure S1).

**Table 1:** The characteristics of study participants

	Total (*N* = 1030)	Samples with both baseline and follow-up visit (*N* = 294)	
		Baseline	Follow-up
Age	49.7 (12.0)	50.31 (9.89)	54.95 (9.83)
Zygosity			
MZ	734 (71.3%)	176 (59.9%)	
DZ	296 (28.7%)	118 (40.1%)	
Sex			
Male	692 (67.2%)	177 (60.2%)	
Female	338 (32.8%)	117 (39.8%)	
Education			
Under middle	223 (30.4%)	119 (40%)	
Middle school	368 (50.1%)	129 (44%)	
Over middle	143 (19.5%)	46 (16%)	
Region			
North	778 (75.5%)	293 (99.7%)	
South	252 (24.5%)	1 (0.3%)	
History of chronic disease			
No	988 (95.9%)	282 (95.9%)	282 (95.9%)
Yes	42 (4.1%)	12 (4.1%)	12 (4.1%)
Alcohol			
No	559 (54.3%)	147 (50.0%)	195 (66.3%)
Yes	471 (45.7%)	147 (50.0%)	99 (33.7%)
LE8 score	68.69 (11.43)	69.68 (11.78)	68.26 (10.94)
Health factor score	78.24 (13.83)	78.27 (14.25)	78.11 (13.15)
Health behavior score	59.13 (16.54)	61.10 (16.53)	58.41 (16.11)

Data are presented as n (%) or mean (standard deviation)

### Association between LE8 and epigenetic clock

In cross-twin analyses, GrimAA, DamAA and DunedinPACE were associated with LE8 scores. After adjusting for sex, age, region, education, history of chronic disease and alcohol use, each 1-point increment in the LE8 score was associated with a decrease of 0.040 years in GrimAA (95%CI: -0.058 to -0.022; *P*: <0.001), a decrease of 0.042 years in DamAA (95%CI: -0.065 to -0.019; *P*: <0.001), and a reduction of 0.002 units in DunedinPACE (95%CI: -0.002 to -0.001; *P*: <0.001)(Table S1). These associations remained significant after excluding participants with chronic diseases (Table S4).

Then, within MZ pairs, we observed significant associations between LE8 and both DamAA and DunedinPACE. Each 1-point increment in the LE8 score was associated with a decrease of 0.039 years in DamAA (95%CI:-0.074 to -0.004; *P* = 0.028) and a decrease of 0.001 units in DunedinPACE (95%CI:-0.002 to -0.000; *P* = 0.022) in Model3 (Table S1).

Table 2 presents the results of the cross-twin analyses and within-MZ twin pairs analyses in Model 3. In both analyses, LE8 score was inversely associated with DamAA and DunedinPACE.

**Table 2:** Association between LE8 score and epigenetic clock

Epigenetic age	Cross-twin^a^		Within-MZ-pair^b^	
	β(95%CI)	*P*	β(95%CI)	*P*
GrimAA	-0.040 (-0.058, -0.022)	**< 0.001**	-0.024 (-0.054, 0.005)	0.104
DamAA	-0.042 (-0.065, -0.019)	**< 0.001**	-0.039 (-0.074, -0.004)	**0.028**
DunedinPACE	-0.002 (-0.002, -0.001)	**< 0.001**	-0.001 (-0.002, -0.000)	**0.022**

Significant results with *P* < 0.05 were highlighted in bold font

^a^We adjusted for sex, age, region, education, history of chronic disease and alcohol use.

^b^We adjusted for region, education, history of chronic disease and alcohol use.

### Association between health/health factors and epigenetic clock

We further examined the associations between the subscales of LE8 (health factor and health behavior) and epigenetic clocks.

In cross-twin analyses, we found that both health factors and health behaviors were negatively associated with DamAA and DunedinPACE. Specifically, each 1-point increase in the health factor score was associated with a mean reduction of 0.027 years in DamAA (*P* = 0.003) and a decrease of 0.001 units in DunedinPACE (*P <* 0.001); each 1-point increase in the health behavior score was associated with a mean decrease of 0.018 years in GrimaAA (*P* = 0.025) and a reduction of 0.001 units in DunedinPACE (*P <* 0.001). GrimAA ($\:{\upbeta\:}$: -0.047; *P* < 0.001) showed a negative association only with health behaviors (Table S1). These associations remained significant after excluding participants with chronic diseases (Table S4).

In within-MZ-pair analyses, the associations between health factors and DunedinPACE ($\:{\upbeta\:}$= -0.001; *P* = 0.012), as well as between health behaviors and GrimAA ($\:{\upbeta\:}$: -0.031; *P* = 0.001), remained significant after controlling for the genetic confounding (Table 3).

**Table 3:** Association between health behavior/health factors and epigenetic clock

LE8 subscale	Epigenetic age	Cross-twin^a^		Within-MZ-pair^b^	
		β(95%CI)	*P*	β(95%CI)	*P*
health factor	GrimAA	0.014 (-0.001, 0.028)	0.062	0.019 (-0.005, 0.042)	0.116
	DamAA	-0.027 (-0.045, -0.009)	**0.003**	-0.023 (-0.051, 0.005)	0.110
	DunedinPACE	-0.001 (-0.001, -0.001)	**< 0.001**	-0.001 (-0.002, -0.000)	**0.012**
health behavior	GrimAA	-0.047 (-0.059, -0.034)	**< 0.001**	-0.031 (-0.050, -0.013)	**0.001**
	DamAA	-0.018 (-0.033, -0.002)	**0.025**	-0.018 (-0.040, 0.005)	0.127
	DunedinPACE	-0.001 (-0.001, -0.001)	**< 0.001**	-0.000 (-0.001, 0.000)	0.349

Note: Significant results with *P* < 0.05 were highlighted in bold font

^a^We adjusted for sex, age, region, education, history of chronic disease and alcohol use.

^b^We adjusted for region, education, history of chronic disease and alcohol use.

### Sex- and age-specific associations between LE8 score and EAA

**Fig. 2:**
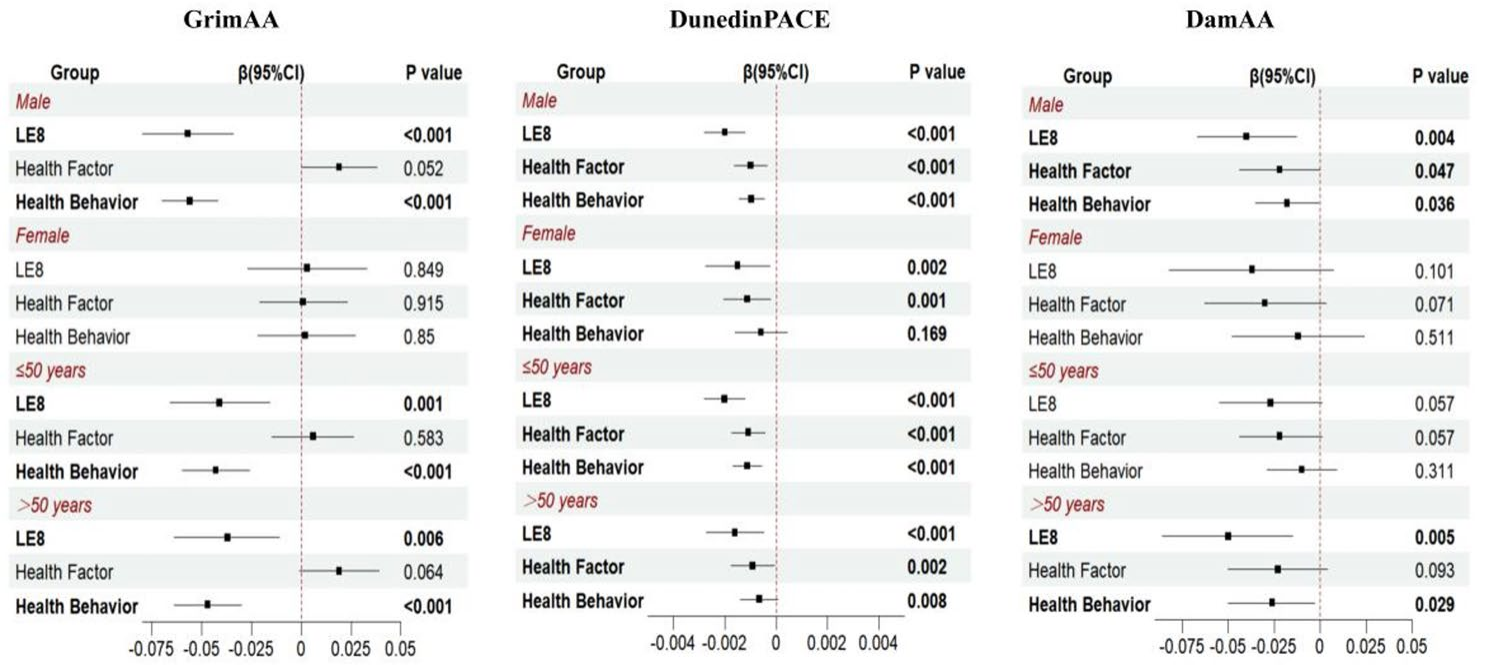
Stratified Analysis of LE8 Score and epigenetic age. We adjusted for sex, age, region, education, history of chronic disease and alcohol use.

Figure 2 presents the result of stratified analyses of the associations between the LE8 total score, its two subscale scores (health behaviors and health factors), and epigenetic aging metrics. For DunedinPACE, higher LE8 total scores and higher scores in both subscales were significantly associated with slower epigenetic aging across all subgroups. GrimAA was significantly associated only with the overall LE8 score and the health behavior subscale, and these associations were observed exclusively among males. For DamAA, significant associations were found with the overall LE8 score as well as both subscale scores; however, these associations were limited to males and individuals aged ≥ 50 years (Table S2). In the interaction analyses, a significant sex × LE8 interaction was observed in the associations of LE8 and health behavior with GrimAA, whereas the age × LE8 interaction was not significant (Table S2).

### Cross-lagged analysis for LE8 and epigenetic age

**Fig. 3:**
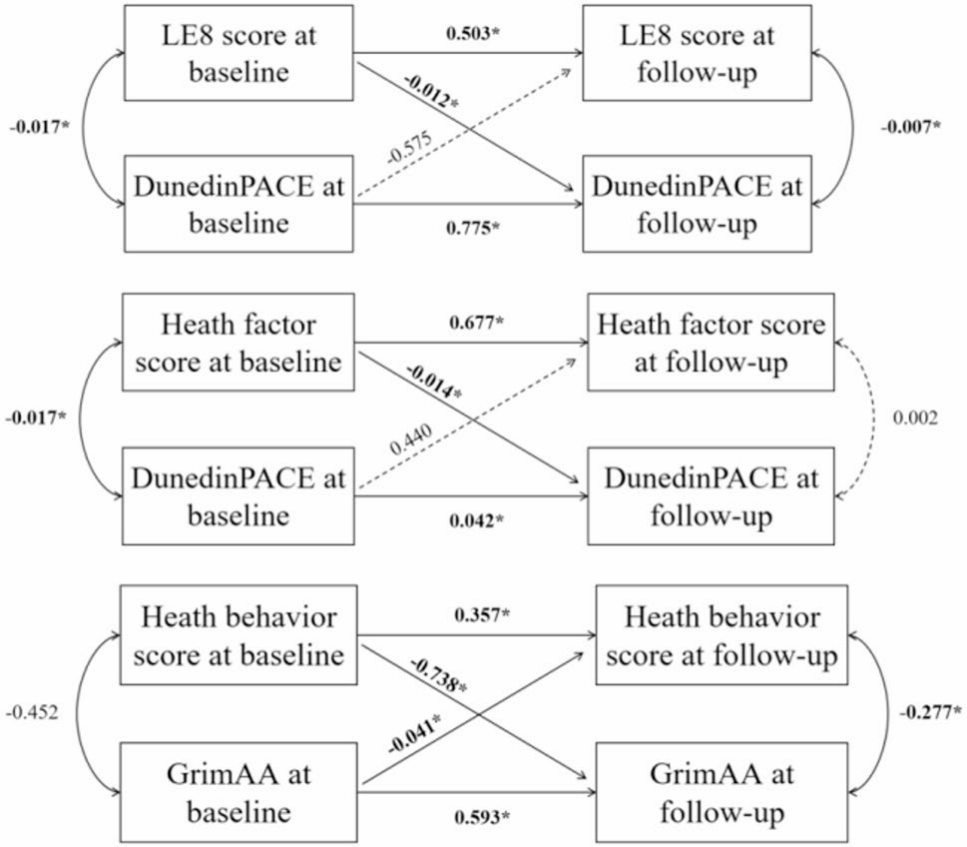
Cross-lagged relationships between LE8 and epigenetic age. Significant results with *P* < 0.05 were highlighted in bold font.

Figure 3 showed the result of the cross-lagged analysis. Across twins, we found that baseline LE8 scores and health factor scores demonstrated significant negative associations with follow-up DunedinPACE. Similarly, baseline health behavior scores showed significant negative associations with follow-up GrimAA. These relationships remained significant when examined specifically within monozygotic (MZ) twin pairs.(Table S3).

## Discussion

In this study, we identified significant negative associations between LE8 and both DunedinPACE and DamAA, between health factor and DunedinPACE, and between health behavior and GrimAA. These negative associations–specifically between LE8 and DunedinPACE, between health factor and DunedinPACE, and between health behavior and GrimAA–were confirmed in cross-lagged analyses.

A relationship between LE8 and aging has been observed in previous studies. Zeng et al. [19] found LE8 and its subscale scores were positively associated with serum klotho levels in the middle-aged and older populations. Klotho is widely acknowledged as a vital protein in the fight against aging, as it plays a significant role in regulating oxidative stress and signaling pathways that are linked to the aging process [20]. Based on the 18,817 participants from UK Biobank data, Li et al. found higher LE8 scores were associated with delayed white matter brain ageing [21].

Emerging evidence has explored the association between DNA methylation, a critical biomarker of biological aging, and LE8. In the Framingham Heart Study involving 5682 participants, Carbonneau et al. [14] found that a 1-SD increase in the LE8 score (13 points) was associated with 0.39-SD lower DunedinPACE and 0.42-SD lower GrimAge. This conclusion is consistent with our cross-twin analyses results. However, in the within-MZ-pair analyses, the association between LE8 and GrimAA was no longer significant, indicating that the association between LE8 and GrimAA may be confounded by genetic factors and shared early-life environmental factors, though the lower power of within-twin analysis can partly explain the loss of significance. Nevertheless, we found that higher LE8 score was associated with slower DunedinPACE and DamAA in both cross-twin analyses and within-MZ-pair analyses, demonstrating robust links between LE8 score and epigenetic aging. Furthermore, in the cross-lagged analysis, we found that higher baseline LE8 scores were associated with lower subsequent DunedinPACE, indicating that better cardiovascular health at baseline may temporally precede and contribute to a slower pace of biological aging over time. We did not find any study that had examined the longitudinal association between LE8 and EAA. Joyce [22] et al. identified an inverse relationship between LS7 (LE8’s precursor) and GrimAge acceleration in the CARDIA cohort. Yet, LE8 differs from LS7 by including a sleep component and modifying factor scoring, limiting direct comparisons. In addition, compared with GEE, the cross-lagged panel model is more suitable for investigating temporal ordering and directional associations between variables. In conclusion, our study contributes to the evidence linking LE8 with aging and suggests that DNA methylation may be one of the underlying mechanisms.

LE8 comprises two subscales: health factor and health behavior. In our study, higher health factor score was associated with lower DunedinPACE, while higher health behavior score was associated with lower GrimAA. Two previous studies based on NHANES data [11, 12] similarly found significant associations between both health behaviors and health factors with PhenoAA (a second-generation clock), with the negative association being stronger for health factors [12]. The differential associations across subscales may reflect distinct biological pathways linking cardiovascular health to aging. Specifically, health behaviors (diet, smoking, physical activity and sleep), primarily influence aging through inflammation, oxidative stress, and neuroendocrine regulation. As the GrimAge clock includes a DNAm-based smoking surrogate, it is particularly sensitive to behavioral and environmental exposures, which may explain its stronger association with the health behavior subscale [23]. In contrast, health factors (blood pressure, glucose, cholesterol, and BMI), reflect cumulative physiological responses to behaviors and genetic predispositions, capturing the body’s current metabolic and cardiovascular status, and are thus more likely to be detected by DunedinPACE, which estimates the rate of physiological aging and is particularly sensitive to such dysregulation [24].

In age- and sex-specific analyses, we observed stronger associations between LE8 and EAA in males in both interaction and stratified analyses, while the age × LE8 interaction was not statistically significant but showed a trend toward stronger associations between LE8 and DamAA among older participants. We did not find any studies that have examined sex and age differences in the relationship. However, several studies investigating the associations between LE8 components and epigenetic aging have reported similar findings. Foster’s study found that BMI was associated with higher epigenetic age in men but not in women [25]. Our previous research also found that the positive associations between epigenetic age metrics and glycemic traits were only present in males [26]. Nevalainen et al. [27] examined the association between BMI and epigenetic age in three age groups (15–24, 40–49 and 90 years), and found a significant association only in the 40–49 age group. These sex- and age-specific associations may reflect biological and behavioral mechanisms. Males generally exhibit higher insulin resistance, fasting glucose, and visceral adiposity [28], which may accelerate aging, making EAA more sensitive to variations in LE8. In contrast, females, especially premenopausal women, benefit from the protective effects of estrogen, which enhance insulin sensitivity and promote a more favorable fat distribution, and a lower overall metabolic burden, resulting in lower metabolic burden and attenuated changes in EAA [28]. Furthermore, studies [23, 29] have shown that epigenetic aging markers show robust and consistent male-biased vulnerability, suggesting that male EAA may be more responsive to exposures. Behavioral differences between sexes, such as smoking, diet, and physical activity, may further contribute; given that GrimAA incorporates DNAm-based smoking pack-years, the higher prevalence of smoking among men could partly explain the stronger LE8–GrimAA association observed in males. Additionally, the smaller number of females in the study population and their higher baseline LE8 scores may also partly explain why the association is less pronounced in females. Age-related differences may further reflect cumulative exposure to unhealthy lifestyles and cardiometabolic stress, along with a reduced capacity for physiological resilience in older adults. In our study, the association between LE8 and DamAA, which captures CpG sites related to detrimental changes [10], appeared stronger in older individuals, supporting this notion.

Aging is an extremely complex and heterogeneous process that affects various cellular and organ functions. These findings advocate for a nuanced approach in future research to better understand how gender and age differences influence CVH and epigenetic aging, emphasizing the importance of personalized interventions in aging and disease prevention strategies.

Our study has several strengths. First, DunedinPACE and GrimAA are widely recognized applied in aging research, allowing for comparison with other research findings. Additionally, this is the first study to apply DamAA in a Chinese twin cohort, providing evidence for its application in the Chinese population. Secondly, we used co-twin control analysis to identify whether shared genetic factors confounded the associations observed. Thirdly, our longitudinal design provides stronger causal inference linking LE8 scores to epigenetic aging. However, our study was also subject to potential limitations. The subjects in this study were from CNTR, a national register of twins recruited as volunteers, they were not representative of the Chinese population. Moreover, LE8 information was collected via self-reported questionnaires, which may introduce bias. Future studies with larger and more representative samples are needed to further explore the relationship between LE8 and aging.

## Conclusion

In conclusion, our study provides novel evidence that higher LE8 score is significantly associated with slower DunedinPACE and DamAA, and this association is robust after controlling for genetic factors. The observed age- and sex-specific differences further emphasize the need for personalized aging and prevention strategies. Our findings, together with prior evidence, support the utility of LE8 as a promising metric of cardiovascular and biological health and underscore the importance of maintaining optimal cardiovascular health to promote healthy aging.

## Supplementary Information

Below is the link to the electronic supplementary material.

Supplementary Material 1

Supplementary Material 2

Supplementary Material 3

## Data Availability

The data that support the findings of this study are available from the corresponding author upon reasonable request.

## Abbreviations

Abbreviation: Meaning
LE8: Life’s essential 8
MZ: Monozygotic twins
DZ: Dizygotic twins
EAA: Epigenetic age acceleration
EA: Epigenetic age
