# Supplementary material for: Association between cardiovascular health and epigenetic aging: a twin study
Source: Clin Epigenetics. 2026 Jan 5;18:23. doi: 10.1186/s13148-025-02034-4 (PMC12870025; doi:10.1186/s13148-025-02034-4)
Supplement: Supplementary file 2 — Supplementary Material 2 [file 13148_2025_2034_MOESM2_ESM.docx]

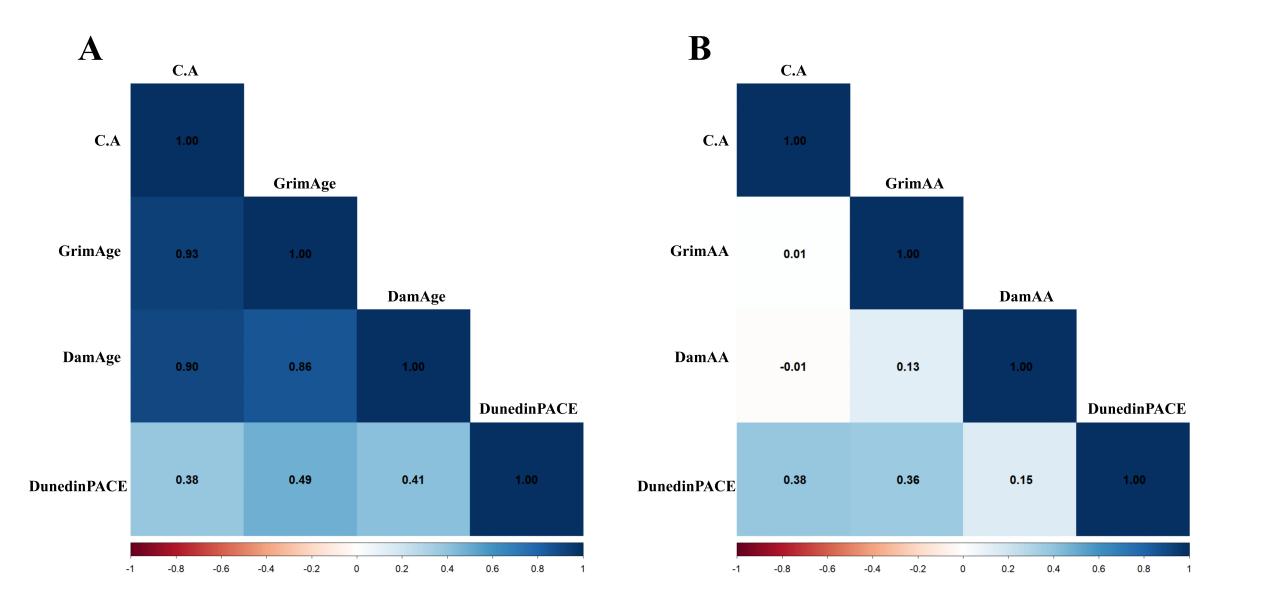


**Supplemental Figure 1**. Correlation Matrix Heatmap of epigenetic age metrics and chronological age

(A) Correlation Matrix of chronological age with epigenetic age.

(B) Correlation Matrix of chronological age with epigenetic age acceleration.

Values indicate Pearson correlation coefficients; color intensity represents effect magnitude.

**Abbreviations:** C. A, chronological age; GrimAA, GrimAge acceleration; DamAA, DamAge acceleration
